# Polyester Vascular Graft Material and Risk for Intracavitary Thoracic Vascular Graft Infection^1^

**DOI:** 10.3201/eid2610.191711

**Published:** 2020-10

**Authors:** Tiziano A. Schweizer, Srikanth Mairpady Shambat, Vanina Dengler Haunreiter, Carlos A. Mestres, Alberto Weber, Francesco Maisano, Annelies S. Zinkernagel, Barbara Hasse

**Affiliations:** University Hospital Zurich, Zurich, Switzerland (T.A. Schweizer, S. Mairpady Shambat, V. Dengler Haunreiter, C.A. Mestres, F. Maisano, A.S. Zinkernagel, B. Hasse);; HerzZentrum Hirslanden Zurich, Zurich (A. Weber)

**Keywords:** Prosthetic vascular graft infection, biofilm, coating, VASGRA, bacteria, gelatin-coated, collagen-coated, polyester

## Abstract

Prosthetic vascular graft infections of the thoracic aorta are rare but can be fatal. Our comparison of collagen- and gelatin-coated grafts showed that collagen-coated grafts were associated with increased biofilm formation and bacterial adherence in vitro and with higher rates of perioperative vascular graft infections in vivo.

Prosthetic vascular graft infections (PVGIs) of the thoracic aorta occur in 1%–3% of patients, but lethality rates are >20% (*1*,*2*). Because of an aging population with multiple medical conditions, more vascular grafts are being implanted, resulting in more PVGIs. Infection often occurs during the perioperative period (*3*) as a consequence of inoculation with bacteria mostly originating from the patient’s own skin flora. PVGIs are biofilm-associated infections in which the matrix around the bacteria impairs chances of treatment success (*4*). Hence, the primary aim is to prevent perioperative infections by identifying risk factors, such as type of prosthesis. 

The few comparative studies reported have focused mostly on use of antibiotic-bonded grafts to reduce the risk for PVGIs in vitro and in vivo (*5*–*7*). However, lack of approval by regulatory authorities, reduced commercial availability, and lack of long-term follow-up data on infection-free survival should be considered (*8*–*10*). Furthermore, selection pressure from the use of topical antibiotics might lead to resistance. *Staphylococcus aureus* has been shown to colonize rifampin-bonded grafts 7 days after implantation (*10*). Hence, implanted grafts are usually coated with proteinaceous solutions only, allowing for quick integration into host tissue. We compared susceptibility of 2 graft materials to biofilm formation in vitro and rates of infections in vivo. 

For our in vitro study, we compared the susceptibility of 2 thoracic vascular woven polyester grafts with different coatings—collagen (collagen graft, InterGard Hemabridge, https://www.getinge.com) and gelatin (gelatin graft, Terumo Aortic, Gelweave, https://terumoaortic.com)—to biofilm formation. The collagen graft is coated with a highly purified form of cross-linked bovine type 1 collagen. The gelatin graft is coated with a modified mammalian gelatin. Gelatin is derived mainly from type 1 collagen by heat denaturation, a process during which collagen loses its native triple helical structure. The resorption time for collagen is 4–8 weeks and for gelatin, 14 days. For our in vivo study, we investigated the rate of infections associated with the 2 grafts among prospective patients undergoing open-chest cardiac surgery at the University Hospital Zurich (Zurich, Switzerland).

## The Study

For the in vitro experiments, we dissected the grafts into 5 × 5 mm square pieces and inoculated them with bacterial strains representing pathogens implicated in thoracic PVGI in our patient cohort. These were derived from either Vascular Graft Cohort Study (VASGRA) patients or laboratory strains (Appendix Table 1, Figure 1) and maintained on Columbia blood agar plates (bioMérieux SA, https://www.biomerieux.com) and in tryptic soy broth (Becton Dickinson, https://www.bd.com) at 37°C. Graft patches were incubated with bacteria in tryptic soy broth–glucose solution (glucose concentration 8 mmol/L) at 37°C for 72 h, and medium was exchanged every 24 h. The patches were washed with phosphate-buffered saline (PBS), sonicated at 44 khz, and the resulting optical density at a wavelength of 600 was measured in a microplate reader. All bacteria, apart from *Pseudomonas aeruginosa* strain 2, showed increased biofilm formation on the collagen graft compared with the gelatin graft patches (Figure, panel A).

**Figure:**
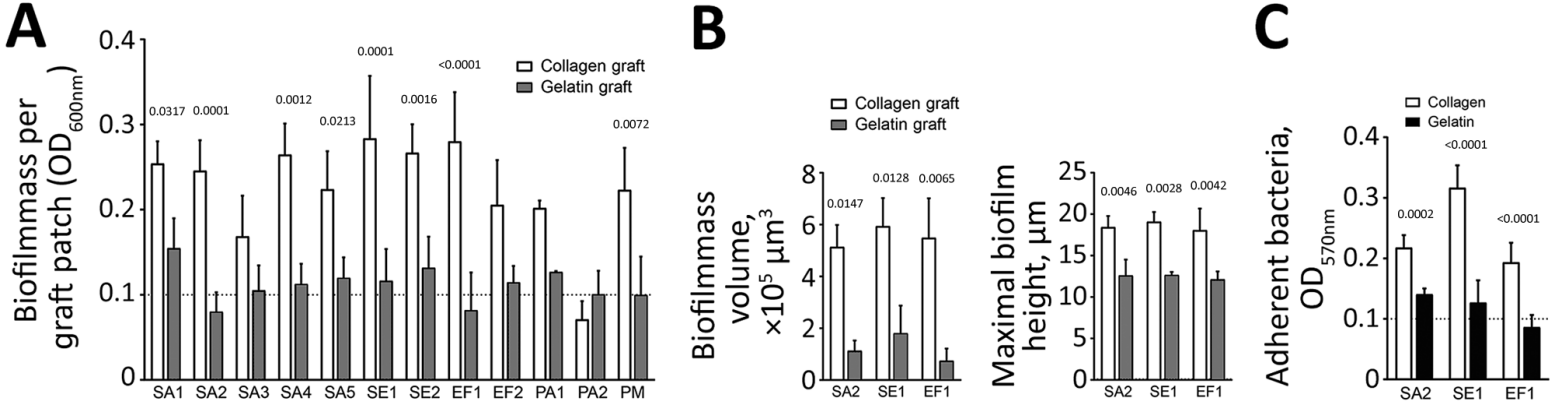
Increased susceptibility of collagen graft to biofilm formation compared with gelatin graft. Graft patches were inoculated with indicated bacterial strains for 72 h and analyzed quantitatively and qualitatively. A) Biofilm formation on the graft patches determined by optical density measurements. B) Total biofilm mass volume and maximal biofilm height, respectively, formed on the graft patches by the 3 clinical isolates—SA2, SE1, and EF— determined from the confocal laser scanning microscopy images with imaris software (https://imaris.oxinst.com). C) Adherence assay to the 2 different coatings used by the grafts. The limit of reliable detection of the plate reader is indicated by the dashed line ( OD_600nm_ = 0.1). All data represent mean ± SD of 3 biological replicates performed in at least 2 technical replicates and were analyzed by using GraphPad Prism 8 (GraphPad Software, https://www.graphpad.com). The values above the graphs represent p values, calculated by using 2-way analysis of variance with the Sidak multiple comparison to determine statistical significance between the 2 graft types or coatings (panels A–C). EF, *Enterococcus faecalis*; OD_600nm_, optical density at a wavelength of 600; PA, *Pseudomonas aeruginosa*; PM, *Pasteurella multocida*; PVGI, prosthetic vascular graft infection; SA, *Staphylococcus aureus*; SE, S*. epidermidis*.

Biofilms of selected strains were stained with SYTO 9 of the LIVE/DEAD *Bac*Light Bacterial Viability Kit (ThermoFisher Scientific, https://www.thermofisher.com) according to the manufacturer’s instructions. The graft patches were placed in 8-well microslides (ibidi, https://ibidi.com) and visualized by confocal laser scanning microscopy with a Leica TCS SP8 inverted microscope (https://www.leica-microsystems.com) under a 63×/1.4 oil immersion objective. We selected 2 representative spots per graft patch, providing a stack of horizontally acquired images (512 × 512 pixels representing an area of 244.8 μm × 244.8 μm) with a z-step size of 0.12 μm. We processed the obtained stacks by using Imaris 9.2.0 software (Bitplane; Oxford Instruments, https://imaris.oxinst.com/support/imaris-release-notes/9-2-0). Biofilm height and volume were determined as previously described (*11*). This approach illustrated the increased biofilm formation on collagen graft patches (Appendix Figure 2). Quantitative analysis from the obtained confocal laser scanning microscopy images corroborated the initial findings because biofilm grown on collagen graft patches displayed increased total biofilm mass volume as well as maximal biofilm height (Figure, panel B).

One possible explanation for the increased susceptibility to biofilm formation could be the distinct coatings of the grafts. Hence, we coated well plates overnight at 4°C with either rat tail collagen 1 (10 μg/mL; ThermoFisher Scientific) or type B gelatin solution (10 μg/mL; Sigma-Aldrich, https://www.sigmaaldrich.com). The plates were incubated with bacteria at 37°C for 30 min. Bacteria were washed, stained with 0.1% crystal violet (Fluka; Sigma Aldrich, https://www.sigmaaldrich.com), solubilized in 95% ethanol, and the resulting optical density at 570 nm was measured. The tested strains adhered substantially better to collagen (Figure, panel C). Our findings are supported by studies demonstrating the potential of gram-positive bacteria to adhere to collagen, whereas only minor affinity was observed for gelatin (*12*,*13*).

To assess the effects of these findings in vivo, we analyzed 412 prospective participants with woven polyester grafts: 28 VASGRA participants in whom intracavitary thoracic PVGI developed and 384 contemporaneous open-chest cardiac surgery patients in whom PVGI did not develop (controls) (Table). Data and strains isolated from patients were used in accordance with Cantonal Ethics Committee approval KEK-ZH-Nr. 2012-0583. For statistical analyses with GraphPad Prism 8 (GraphPad Software, https://www.graphpad.com), we used nonparametric tests (Fisher exact or Wilcoxon rank-sum, as appropriate). When normalized to the total number of control patients (n = 384 who had undergone the cardiac surgery but had no PVGI), the calculated percentage of intracavitary thoracic PVGI (n = 28 VASGRA patients who had undergone the cardiac surgery and had PVGI) was higher for patients in the collagen-graft (10.8%) versus the gelatin-graft group (3.52%; p<0.005).

**Table:** Risk for early and late intracavitary thoracic PVGI among cardiac surgery patients with vascular woven polyester grafts coated with collagen or gelatin, Zurich, Switzerland*

Demographics and risk factors	Early thoracic PVGI, n = 15	Late thoracic PVGI, n = 13	Cardiac surgery, no PVGI, n = 384	p value†
Demographic				
Sex				0.27
M	3 (20)	3 (23)	54 (14)	
F	12 (80)	10 (77)	330 (86)	
Caucasian race	15 (100)	12 (92)	375 (98)	0.51
Age, y, median (IQR)	65 (54–68)	65 (51–68)	58 (49–67)	0.89
Risk factors				
BMI, kg/m^2^, median (IQR)	27 (24–33)	26 (23–28)	26 (24–29)	0.97
Smoking				0.69
Never	6 (40)	6 (46)	150 (39)	
Former/current	9 (60)	7 (54)	234 (61)	
Alcohol consumption	4 (27)	3 (23)	131 (34.0)	0.41
Hypertension	12 (80)	8 (62)	273 (72)	1.0
Diabetes mellitus	2 (13)	1 (8)	54 (14)	0.78
Dyslipidemia	7 (47)	6 (46)	196 (51)	0.70
Cardiac events	8 (53)	7 (54)	173 (45)	0.43
Cerebrovascular events	3 (20)	3 (23)	58 (15)	0.41
Coagulopathy	0 (0)	0 (0)	4 (1.0)	1.0
Malignancy,	4 (27)	2 (15)	69 (18)	0.62
Charlson Comorbidity Index score, median (IQR)	2 (1–5)	2 (1–4)	1 (1–3)	0.27
Index surgery				0.55
Dissection	7 (57)	6 (46)	154 (40)	
Aneurysm/pseudoaneurysm	8 (53)	7 (54)	230 (60)	
Symptoms at index operation				0.004
Asymptomatic	4 (27)	5 (23)	223 (58)	
Symptomatic	9 (60)	10 (77)	161 (42)	
Setting of index operation				0.08
Elective	9 (60)	7 (54)	284 (74)	
Urgent/emergency	6 (40)	6 (46)	100 (26)	
ASA class at index operation				<0.001
Grade <III	7 (47)	3 (23)	38 (10)	
Grade >III	8 (53)	10 (77)	346 (90)	
Cardiopulmonary bypass time, min, median (IQR)	174 (154–338)	163 (84–363)	206 (154–338)	0.23
Polyester vascular woven grafts				0.005
Collagen graft	11 (74)	9 (69)	165 (39.5)	
Gelatin graft	4 (27)	4 (31)	219 (52.4)	
PVGI				
Assumed route				NA
Perioperative	15 (100)	0	NA	
Hematogenous	0	8 (62)	NA	
Contiguous	0	5 (38)	NA	
Microorganisms				NA
* Staphylococcus aureus*	4 (27)	3 (23)	NA	
Coagulase-negative staphylococci	2 (13)	1 (7.7)	NA	
* Enterococcus faecalis*	0	2 (15)		
* Streptococcus* spp	4 (27)	0	NA	
* Cutibacterium acnes*	1 (7)	2 (15)	NA	
* Pseudomonas aeruginosa*	1 (7)	0	NA	
* Pasteurella multocida*	1 (7)	0	NA	
Culture negative	2 (14)	3 (23)	NA	

*Values are no. (%) unless otherwise indicated. ASA, American Society of Anesthesiology; BMI, body mass index; IQR, interquartile range; NA, not applicable; PVGI, prosthetic vascular graft infection. †p values from Fisher exact test (categorical variables) and Wilcoxon rank-sum test (continuous variables) comparing patients with PVGI (early and late combined) and patients without PVGI.

## Conclusions

We found more biofilm formation on collagen-coated polyester vascular grafts than on gelatin-coated grafts, possibly because the tested strains adhered substantially better to collagen than to gelatin. When we analyzed prospective patients with PVGI and contemporary controls, the percentage of PVGI associated with collagen-coated grafts was also higher.

The strengths of our study include use of patient-derived strains of bacteria and the prospective collection of patients with incident PVGIs and controls. Our study has some limitations. First, it was a single-center study, resulting in low patient numbers, and the role of the graft material as a risk factor for PVGI is difficult to prove because of the rarity of the infection. Furthermore, data for patients with PVGI have to be interpreted with caution because some patients have additional foreign material in the heart. Second, publicly available information on the exact type of coating as well as the application procedures used is lacking. In addition, the experiments were performed in a static experimental setup instead of a flow chamber. However, because we were interested in direct bacterial adherence to the graft material, simulating a scenario in which infection would occur as consequence of unintentional inoculation during the perioperative period, we believe that the setup used is adequate. We did not reproduce a gram-positive and gram-negative mixed biofilm formation experiment because a monomicrobial setup enabled us to determine which material and coating was more susceptible to bacterial adherence in a more controllable fashion.

In conclusion, biofilm formation was increased on collagen-coated vascular grafts compared with gelatin-coated grafts in vitro. As opposed to another risk factor analysis from the VASGRA study (*3*), in our study, the graft material was associated with the PVGI rate. Parameters such as vascularization potential, secure pseudointima growth, and reduced thrombogenicity are perceived as affecting successful integration and functionality of prosthetic vascular grafts (*14*). Further parameters should be considered in the future design and development of vascular prostheses to reduce the emerging trend of PVGI.

Appendix Additional materials and results for study of polyester vascular graft material as risk factor for intracavitary thoracic vascular graft infection.
