# Supplementary material for: Polyester Vascular Graft Material and Risk for Intracavitary Thoracic Vascular Graft Infection
Source: Emerg Infect Dis. 2020 Oct;26(10):2448–52. doi: 10.3201/eid2610.191711 (PMC7510746; doi:10.3201/eid2610.191711)
Supplement: Appendix — Additional materials and results for study of polyester vascular graft material as risk factor for intracavitary thoracic vascular graft infection. [file 19-1711-Techapp-s1.pdf]

Article DOI: https://doi.org/10.3201/eid2610.191711

Polyester Vascular Graft Material and Risk for Intracavitary Thoracic Vascular Graft Infection

Appendix

Appendix Table. Bacterial strains used in this study

Strain	Description	Source (reference)
Staphylococcus aureus		
SA1	Clinical PVGI* isolate	This study
SA2	Clinical PVGI isolate	This study
SA3	Clinical PVGI isolate	This study
SA4	HG003, MSSA†	(*1*)
SA5	JE2 USA300, MRSA‡	NARSA§
Staphylococcus epidermidis		
SE1	Clinical PVGI isolate	This study
SE2	RP62A	(*2*)
Enterococcus faecalis		
EF1	Clinical PVGI isolate	This study
EF2	M10, VRE¶	ATCC 51299™
Pseudomonas aeruginosa		
PA1	Clinical PVGI isolate	This study
PA2	PA01	(*3*)
Pasteurella multocida		
PM	Clinical PVGI isolate	This study

*PVGI, Prosthetic vascular graft infection.
†MSSA, Methicillin-susceptible *Staphylococcus aureus*.
‡MRSA, Methicillin-resistant *Staphylococcus aureus.*
§NARSA, Network on Antimicrobial Resistance in *Staphylococcus aureus*.
¶VRE, Vancomycin-resistant *Enterococcus*.

Strain	Background
S. aureus (SA)	
1 - 3	PVGI isolates
4 - 5	Laboratory strains
S. epidermidis (SE)	
1	PVGI isolate
2	Laboratory strain
E. faecalis (EF)	
1	PVGI isolate
2	Laboratory strain
P. aeruginosa (PA)	
1	PVGI isolate
2	Laboratory strain
P. multocida (PM)	PVGI isolate

Appendix Figure 1. Background information about bacterial strains used in study of polyester vascular graft material and risk for intracavitary thoracic vascular graft infection.

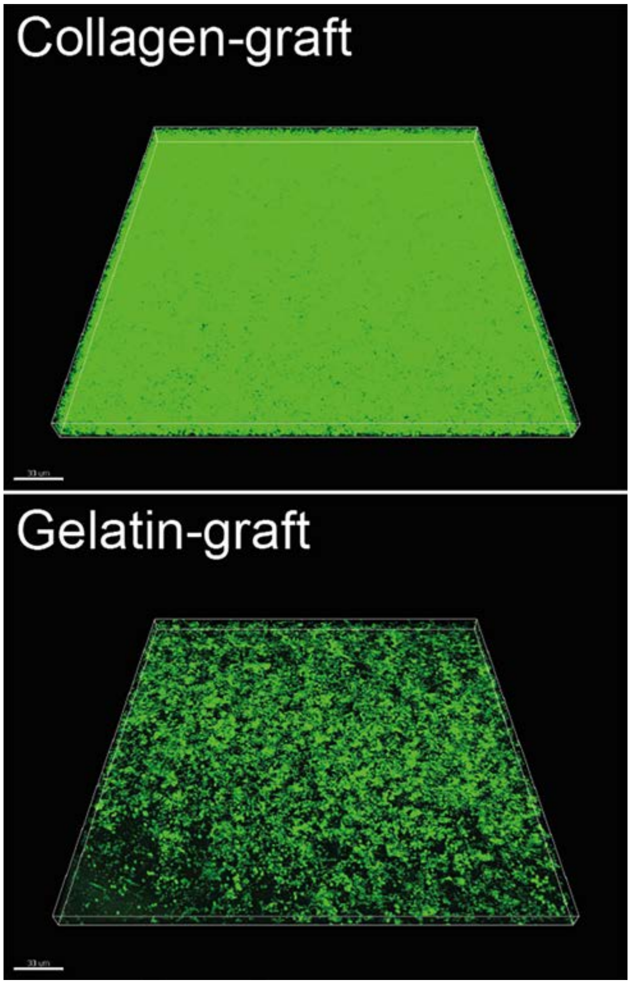

Appendix Figure 2. Representative images of biofilm formation of the clinical isolate *Staphylococcus aureus* 2 on the 2 graft types by confocal laser scanning microscopy. The images correspond to 3-dimensional reconstructions obtained from confocal z-stack images. Scale bars indicate 30 μm.

References

1. Herbert S, Ziebandt AK, Ohlsen K, Schäfer T, Hecker M, Albrecht D, et al. Repair of global regulators in *Staphylococcus aureus* 8325 and comparative analysis with other clinical isolates. Infect Immun. 2010;78:2877–89. PubMed https://doi.org/10.1128/IAI.00088-10

2. König DP, Perdreau-Remington F, Rütt J, Stoβberger P, Hilgers R-D, Plum G. Slime production of *Staphylococcus epidermidis*: increased bacterial adherence and accumulation onto pure titanium. Acta Orthop Scand. 1998;69:523–6. PubMed https://doi.org/10.3109/17453679808997791

3. Holloway BW. Genetic recombination in *Pseudomonas aeruginosa*. J Gen Microbiol. 1955;13:572–81. PubMed
